# Cognition mediates the relation between structural network efficiency and gait in small vessel disease

**DOI:** 10.1016/j.nicl.2021.102667

**Published:** 2021-04-20

**Authors:** Mengfei Cai, Mina A. Jacob, David G. Norris, Marco Duering, Frank-Erik de Leeuw, Anil M. Tuladhar

**Affiliations:** a Radboud University Medical Center; Donders Institute for Brain, Cognition and Behaviour; Department of Neurology, Nijmegen, The Netherlands; b Center for Cognitive Neuroimaging, Donders Institute for Brain, Cognition and Behaviour, Nijmegen, The Netherlands

**Keywords:** Small vessel disease, Gait, Cognition, Network efficiency

## Abstract

Cerebral small vessel disease (SVD), including white matter hyperintensities (WMH), microbleeds, lacunes, was related to gait disturbances, while the underlying mechanism is unclear. Here, we investigated the relation between structural network efficiency, cognition and gait performance in 272 elderly subjects with SVD. All participants underwent 1.5 T MRI, gait and neuropsychological assessment. Conventional MRI markers for SVD, i.e. WMH volume, number of lacunes and microbleeds, were assessed. Diffusion tensor imaging-based tractography was used to reconstruct the brain network for each individual, followed by graph-theoretical analyses to compute the well-established network measure, global efficiency. We found that lower global efficiency was associated with worse gait performance, including slower gait speed and shorter stride length, independent of conventional MRI markers for SVD. This association was partly mediated via cognitive function. We identified subnetworks of white matter connections associated with gait and cognition, characterized by dominant involvement of frontal tracts. Our findings suggest that network disruption is associated with gait disturbances through cognitive dysfunction in elderly with SVD. Gait is a highly cognitive process and the crucial role of cognition should be considered when investigating gait disturbances in the elderly with SVD.

## Highlights

• Lower global efficiency is associated with worse gait performance in SVD.
• This association is partially mediated by cognitive performance.
• Gait is a highly cognitive process in the elderly with SVD.

## 1 Introduction

Gait disturbances are prevalent in the elderly and are associated with adverse consequences, including falls, institutionalization and death (Abellan Van Kan et al., 2009, Cesari et al., 2005, Studenski, 2011). Cerebral small vessel disease (SVD), including conventional SVD markers on MRI, i.e. white matter hyperintensities, microbleeds and lacunes, has been identified as an important contributor to gait disturbances (de Laat et al., 2010). However, these SVD-related lesions alone fail to fully account for gait disturbances (de Laat et al., 2010), indicating other mechanisms, not covered by these conventional SVD markers, might play an important role in gait disturbances.

Gait is a complex sensorimotor function, which is controlled by the widespread brain networks. These networks regulate locomotion and control gait by integration of multisensory information (Reijmer et al., 2013, Takakusaki, 2013, Takakusaki, 2017). Furthermore, gait is strongly related to cognitive function, especially the elderly rely more on cognitive rather than sensorimotor control mechanisms, due to loss of age-related sensorimotor functioning (Takakusaki, 2013, Takakusaki, 2017). These widespread brain networks might be vulnerable to the cumulative effects of multiple spatially distributed SVD-related lesions, potentially contributing to reduced integration of information (e.g. sensory input and motor coordination) coming from different brain regions (Lawrence et al., 2014, Tuladhar et al., 2016). The efficiency of a brain network can be investigated with graph-theory based structural connectivity, as assessed by diffusion tensor imaging (DTI). Global efficiency, a graph-theoretical network measure that reflects the extent to which information communication is globally integrated in a network, is commonly used as a marker of network integrity.^9^ A previous study showed that lower global efficiency was related to slower gait velocity, however, this study had a small sample size and was performed in patients with cerebral amyloid angiopathy (Reijmer et al., 2015). Although global efficiency is strongly related to cognitive function (Lawrence et al., 2014, Tuladhar et al., 2016), it is currently unknown whether global efficiency is related to gait performance and whether this relation is mediated via cognition in elderly with sporadic SVD.

In the present study, we hypothesized that structural network efficiency is associated with gait performance in SVD and that this association is mediated by cognitive function. To this end, we first assessed the relationship between global efficiency and quantitative gait measurements in individuals with SVD. Secondly, we examined the relation between cognition and gait as well as whether cognitive function mediates the relationship between global efficiency and gait. Thirdly, at regional level, we conducted network-based statistic (NBS) to identify the gait- and cognition-related subnetwork and investigated how the investigated subnetworks of white matter connections are related in elderly with sporadic SVD.

## 2 Methods

### 2.1 Study population

This study is part of the Radboud University Nijmegen Diffusion Tensor and Magnetic resonance Cohort (RUN DMC) study, an ongoing longitudinal prospective single-center study that aims to investigate the risk factors and clinical consequences among elderly with sporadic SVD. Consecutive participants referred to the neurology department were included at baseline in 2006. SVD was characterized on neuroimaging by either WMH and/or lacunes of presumed vascular origin. Detailed description of the patient recruitment and study rationale of the RUN DMC study, have been described in the study protocol (van Norden et al., 2011).

At baseline and during two follow-up time points patients underwent a standardized MRI protocol, a comprehensive neuropsychological assessment, physical examination as well as motor and gait assessments. This cross-sectional study is based on data collected during the first follow-up in 2011, due to an optimized MRI protocol in 2011 compared with 2006. Of the 503 participants included at baseline, 398 participated in the follow-up examination. For the present study, we excluded 126 participants, yielding a final sample of 272. Exclusion reasons at follow-up in 2011 are presented in Supplementary Fig. 1.

The Medical Review Ethics Committee Region Arnhem–Nijmegen approved the study, and all participants gave written informed consent.

### 2.2 Cardiovascular risk factors

We assessed the presence of hypertension, smoking, diabetes mellitus and hypercholesterolemia by standardized assessment and questionnaires, as described previously (van Norden et al., 2011). Hypertension was defined as the use of antihypertensive medication, and/or a current (in 2011) systolic blood pressure ≥ 140 mm Hg and/or diastolic blood pressure of ≥ 90 mm Hg.

### 2.3 Gait assessment

Gait performance was assessed by using a 5.6 m electronic, pressure sensitive walkway (GAITRite, MAP/CIR Inc., Havertown, PA, USA), connected to a computer, which has an excellent test–retest reliability and validity (Bilney et al., 2003, Menz et al., 2004) The quantitative GAITRite parameters were averaged over two walks. Participants were instructed to walk over the walkway at their usual/normal gait speed. In order to measure steady-state walking, they were asked to start walking 2 m before the carpet and to stop until 2 m beyond it. The mean values of two trials were measured to obtain reliable gait parameters. The following gait parameters were recorded: gait speed (m/s), stride length (in meters; i.e. distance between the heel points of two consecutive footprints of the same foot), stride width (in meters; i.e. distance between one midpoint of a footprint and the line of progression of the opposite foot) and cadence (steps/minute). We chose to use these parameters since they are commonly used to investigate gait performance in SVD (de Laat et al., 2011a, de Laat et al., 2011b, de Laat et al., 2010). Besides, we also included gait variability with respect to stride time and length and it was calculated as the coefficient of variation in percentage (CV = [standard deviation of parameter/mean of parameter] × 100%). It reflects the magnitude of stride-to-stride fluctuations within one gait parameter, with less variability indicating higher gait automaticity and stability (Finsterwalder et al., 2019, Hausdorff, 2005). Gait impairment was defined as gait speed lower than 1 m/s (van der Holst et al., 2018).

### 2.4 Neuropsychological assessments

Cognitive function was assessed by using a standardized neuropsychological test battery, which included the Mini Mental State Examination (MMSE), Rey Auditory Verbal Learning Test (RAVLT), Rey’s Complex Figure Test (RCFT), Paper-Pencil Memory Scanning Task (PPMST), Stroop test, Verbal Series Attention Test (VSAT) and Symbol-Digit Substitution Task (SDST), Verbal Fluency Tasks. Detailed information has already been published (van Norden et al., 2011).

Performance across tests was made comparable by transforming the raw test scores into z-scores (individual test score minus mean test score, divided by the standard deviation). Z-scores for follow-up (2011) were calculated using the mean and SD of the baseline (2006) tests. Higher z-scores indicate a better performance.

Cognitive index was constructed to evaluate global cognitive function as previously reported (van Uden et al., 2015). Briefly, this was calculated as the mean of the z-scores of the Speed-Accuracy Trade-Off (SAT) score of the 1-letter subtask of the PPMST, the mean of the SDST, the mean of the SAT score of the reading task of the Stroop test, and the mean of the added score on the three learning trials and the mean of the delayed recall of the RAVLT. Compound scores for each specific cognitive domain are described in detail in Supplementary Table 1.

### 2.5 MRI acquisition

MR images were acquired on a single 1.5 Tesla scanner (Siemens, Magnetom Avanto). The protocol included the following whole brain scans: T1-weighted 3D MPRAGE sequence (TR/TE/TI: 2250/2.95/850 ms, isotropic voxel size: 1.0 mm^3^), FLAIR (TR/TE/TI: 14240/89/2200 ms, voxel size: 1.2 × 1.0 × 2.5 mm, interslice gap 0.5 mm), T2*-weighted gradient echo sequence (voxel size: 1.3 × 1.0 × 5.0 mm; interslice gap 1.0 mm) and a DTI sequence (TR/TE: 10200/95, isotropic voxel size: 2.5 mm^3^), 7 unweighted scans, 61 diffusion weighted scans at b = 900 s/mm^2^. Complete acquisition details have been described previously (van Leijsen et al., 2017, van Norden et al., 2011).

### 2.6 Conventional MRI markers of SVD

We segmented WMH semi-automatically using FLAIR and T1 sequences as described previously (Ghafoorian et al., 2016). All segmentations were visually inspected for segmentations errors by one trained rater, blinded for clinical data. WMH volumes were normalized to intracranial volume. Lacunes were manually rated on T1-weighted and FLAIR images, and microbleeds on the T2-weighted images. The rating was performed according to STRIVE criteria (Wardlaw et al., 2013). Both markers were rated by two trained raters (MC and MJ), followed by a consensus meeting (with MD, AT, and FEdL). Grey matter volume (GMV) and white matter volume (WMV) were computed using SPM12 unified segmentation on T1 MPRAGE sequences, and were calculated by summing all voxels belonging to tissue class multiplied by voxel volume (ml). Total brain volume was determined by the sum of GMV and WMV.

### 2.7 DTI analysis

Raw diffusion weighted data were denoised by using a local principal component analyses filter, followed by correction for cardiac and head motion, and eddy currents by using the PATCH algorithm, as described previously (Zwiers, 2010). Susceptibility distortions were unwrapped by normalizing the images to the T1 images in the phase-encoding direction using SPM12. FSL was then used to extract brain tissue and calculate the diffusion tensor. In-house software was used to perform whole brain deterministic tractography by seeding from a 0.5 mm^3^ grid, with streamlines terminated when the angle between principal eigenvectors ≥ 40° or FA < 0.2 (Lawrence et al., 2014).

### 2.8 Structural network construction

We parcellated each subject’s brain into 45 regions per hemisphere using the Automatic Anatomical Label (AAL) template (Tzourio-Mazoyer et al., 2002), excluding the cerebellar regions. For this purpose, T1-weighted images were linearly registered to the b0-image using FMRIB’s Linear Image Registration Tool (FLIRT, part of FSL) and non-linearly registered to Montreal Neurological Institute (MNI) 152 template using ANTs. These transformations were finally combined to register the AAL template to each subject’s diffusion space.

Two regions were considered connected if the endpoints of the reconstructed streamline were located within both regions. A weighted connection (i.e. edge) was computed, where weights were defined based on the sum of the inverse streamline lengths modified from Hagmann et al. (2007) The threshold for weighted edges was set at 1.0 to reduce noise-related false-positive connections (Lawrence et al., 2014). This resulted in a weighted 90x90 connectivity matrix for each participant.

### 2.9 Network measures

We used the brain connectivity toolbox (http://www. brain-connectivitytoolbox.net/) to compute graph-theoretical measures (Rubinov and Sporns, 2010). Structural network measures have a high reproducibility in small vessel disease (Lawrence et al., 2018) We calculated the following 3 core network measures: density, total network strength and global efficiency. Density and network strength are basic network measures, while global efficiency was used to measure the organization of connections. The latter showed the strongest relationship with cognition in SVD (Tuladhar et al., 2016, Tuladhar et al., 2020). Global efficiency is the average inverse shortest path length in the network.

### 2.10 Statistical analysis

All statistical analyses were carried out in R, version 3.5.1 (https:www.r-project.org/) (R Core Team, 2016). Two-tailed p values < 0.05 were considered statistically significant.

The baseline characteristics were presented as mean ± standard deviation (SD) for normally distributed data, median and interquartile ranges (IQR) for the skewed distributed parameters. Normalized WMH volume was log-transformed to obtain normal distribution.

Firstly, we performed linear regression analysis to examine the relationship between global efficiency and gait parameters and between cognitive domains and gait parameters, adjusted for potential confounders (age, gender, height, number of lacunes and microbleeds, white matter hyperintensity volume, total brain volume). Correction for multiple testing (i.e., five) was performed via the Bonferroni method.

Secondly, we tested whether cognitive function mediated the relationship between global efficiency and gait parameters. We performed the mediation analysis using ‘lavaan’ (version 0.6.5) with cognitive performance as the mediator (Rosseel, 2012). Using lavaan, we estimated the direct effect of global efficiency on gait and the indirect effect of global efficiency on the gait via cognitive index, separately for gait speed and stride length.

Thirdly, we examined which connections are related to gait parameters, we used the Network-Based Statistic (NBS) toolbox (nitrc.org/projects/nbs) (Zalesky et al., 2010) Edges with statistically significant association were defined as t > 2.34 (corresponding to p-uncorrected < 0.01). Multiple comparisons were controlled using family-wise error rate (FWER) and data was permuted 5000 times.

## 3 Results

Table 1 shows demographics, vascular risk factors, imaging and gait characteristics.

**Table 1:** Characteristics of the study population.

n	272
Demographics	
Age, years (mean (SD))	68.4 (8.4)
Sex, male (%)	120 (44.1)
Education, years (mean (SD))	11.46 (3.50)
MMSE score (mean (SD))	28.22 (2.04)
Vascular risk factors	
Hypertension, n (%)	211 (77.9)
Diabetes, n (%)	31 (11.5)
Hypercholesterolemia, n (%)	118 (43.7)
Smoking ever, n (%)	43 (15.9)
BMI, kg/m^2^ (mean (SD))	27.58 (4.17)
Neuroimaging	
Network density (mean (SD))	0.12 (0.02)
Network strength (mean (SD))	17.97 (2.17)
Global efficiency (mean (SD))	10.30 (2.51)
Local efficiency (mean (SD))	10.14 (1.98)
WMH, ml (median [IQR])	2.94 [1.28, 8.89]
Microbleeds, n (%)	56 (20.7)
Lacunes, n (%)	61 (22.5)
Total brain volume, ml (mean (SD))	1085.22 (146.37)
Gait characteristics	
Gait Speed, m/s (mean (SD))	1.18 (0.22)
Stride length, m (mean (SD))	1.26 (0.18)
Cadence, steps/min (mean (SD))	112.89 (9.59)
Stride time variability, %	2.3 (2.01)
Stride length variability, %	1.6 (1.70)
Gait impairment, n (%)	55 (20.2)

Data represent number of participants (%), mean ± SD or median (IQR). WMH: white matter hyperintensity.

### 3.1 Association between global efficiency and gait

We found significant associations between global efficiency and gait speed (β = 0.18; *p* = 0.016), and between global efficiency and stride length (β = 0.23; *p* < 0.001). After additional adjustment for conventional MRI markers for SVD, global efficiency remained significantly associated with gait speed (β = 0.23; *p* = 0.008) and stride length (β = 0.25; *p* < 0.001) (Table 2). No significant association was found between global efficiency and gait cadence, stride time and length variability, either without or with adjustment for conventional MRI markers.

**Table 2:** Association between global efficiency and gait parameters. Data present standardized estimates with corresponding p-values after correction for multiple (i.e., five) comparisons. Model 1: adjustment for age, sex, height; Model 2: additional adjustment for number of lacunes and microbleeds, white matter hyperintensity volume and total brain volume.

Gait parameters	Model 1		Model 2	
	β	*p-*value	β	*p-*value
Gait speed	0.18	0.023	0.23	0.01
Stride length	0.23	<0.001	0.25	<0.001
Gait cadence	0.002	1.00	0.11	0.847
Stride time variability	−0.06	1.00	−0.09	1.00
Stride length variability	−0.04	1.00	−0.14	0.57

### 3.2 Association between cognition and gait

Global cognitive function, reflected by the cognitive index, was significantly associated with all gait parameters except stride time variability, independent of conventional MRI markers for SVD (Table 3).

**Table 3:** Association between cognitive index and gait parameters. Data present standardized estimates with corresponding p-values after correction for multiple (i.e., five) comparisons. Model 1: adjustment for age, sex, height; Model 2: additional adjustment for number of lacunes and microbleeds, white matter hyperintensity volume and total brain volume.

Gait parameters	Model 1		Model 2	
	β	*p-v*alue	β	*p-*value
Gait speed	0.35	<0.001	0.36	<0.001
Stride length	0.32	<0.001	0.32	<0.001
Gait cadence	0.24	0.001	0.28	<0.001
Stride time variability	−0.13	0.313	−0.14	0.294
Stride length variability	−0.19	0.041	−0.21	0.022

**Fig. 1:**
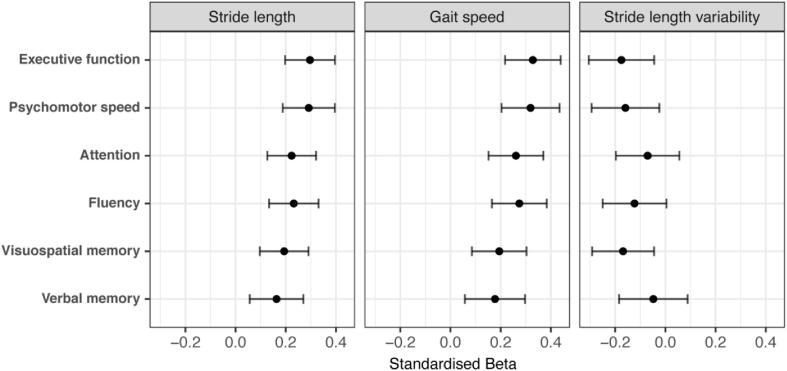
Effect sizes for associations between cognitive domains and gait parameters. Standardized estimates for associations between cognitive domains and stride length, gait speed and stride length variability are presented as point estimate and corresponding 95% confidence interval.

Next, we investigated which cognitive domain had the strongest association with stride length, gait speed and stride length variability. All cognitive domains were associated with stride length, with highest effect sizes for psychomotor speed and executive function. Similar results were found in the relation to gait speed (Fig. 1). Also, stride length variability was associated with psychomotor speed, executive function and visuospatial memory.

### 3.3 Cognition mediates the relation between global efficiency and gait

Given the association between global efficiency and gait as well as between cognition and gait, we examined whether the relation between global efficiency and gait is mediated by cognition.

**Fig. 2:**
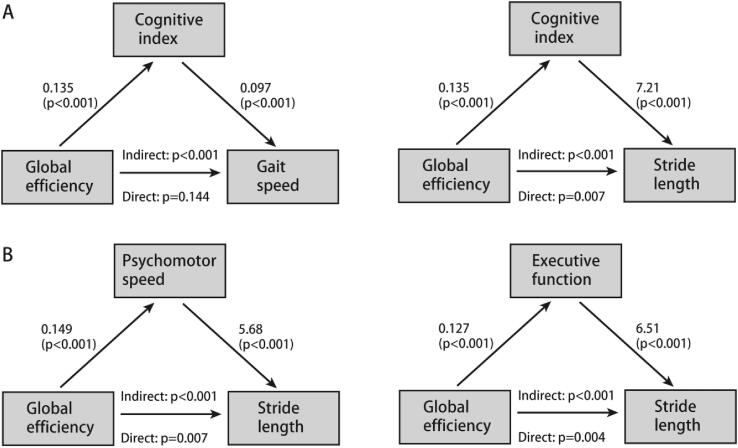
Cognitive functions and domains mediate the association between global efficiency and gait parameters. (A) Mediation models for cognitive function between global efficiency and gait speed and stride length. (B) Mediation models for psychomotor speed and executive function between global efficiency and stride length. Diagrams present standardized coefficients with *p* values for all associations. The statistical significance of direct and indirect paths is presented in the center of the diagram.

Cognitive index fully mediated the relation between global efficiency and gait speed (indirect effect: *p* < 0.001; direct effect: *p* = 0.144), while the relation between global efficiency and stride length was partly mediated via cognitive index (indirect effect: *p* < 0.001; direct effect: *p* = 0.007) (Fig. 2A).

Given that executive function and psychomotor speed are dominantly affected by SVD and based on our findings that cognitive index partially mediated the association between global efficiency and stride length, we further investigated the mediating effect of these two cognitive domains. Both executive function and psychomotor speed partially mediated the association between global efficiency and stride length (Fig. 2B).

### 3.4 Subnetworks associated with gait and cognition

**Fig. 3:**
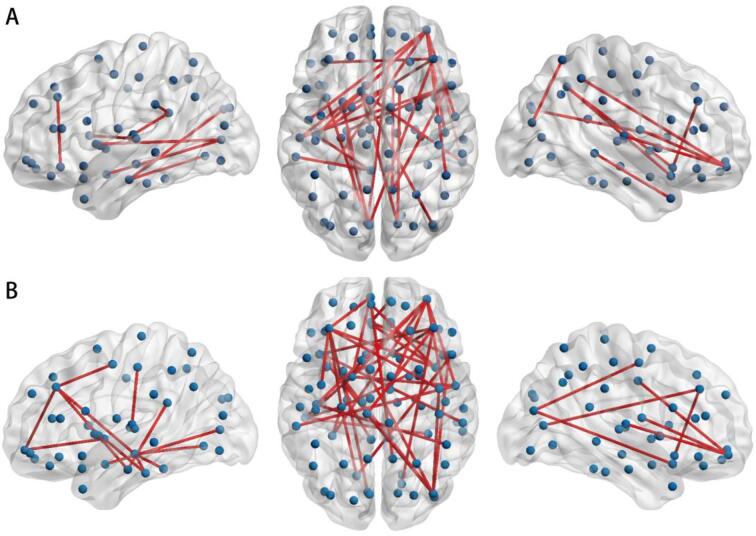
Subnetwork of connections related to gait and cognition. Red lines represent subnetwork of connections associated with stride length (A), controlling for age, sex, height; (B) with cognition controlling for age, sex and education. (For interpretation of the references to colour in this figure legend, the reader is referred to the web version of this article.)

Next, we examined the relation between brain network, gait and cognition at regional level. A subnetwork associated with stride length, predominantly involving the frontal lobe, including fronto-frontal, fronto-occipital, fronto-temporal, fronto-parietal pathways, was observed, while no subnetwork was found for gait speed (Fig. 3A). In addition, a subnetwork associated with cognition was identified with dominant connections involving the frontal lobe as well (Fig. 3B).

As the analyses of the whole brain network connectivity showed that relation between global efficiency and gait is mediated by cognitive function, we examined whether the relation between the white matter connections and gait would alter, if cognitive function was added in the model. This analysis showed that, after adjusting for cognitive index, no single subnetwork was significantly associated with gait (data not shown).

## 4 Discussion

In SVD, lower global efficiency was associated with worse gait performance, a relationship partly mediated by cognition. These findings suggest that network disruption is associated with gait disturbances through cognitive dysfunction in the elderly with SVD. Our results indicate that gait is a highly cognitive process and the crucial role of cognition should be considered when investigating gait disturbances in the elderly with SVD.

Using whole brain network analysis and regional network analysis, we found that cognition mediated the relation between network efficiency and gait performance in SVD. This highlights the cognitive control mechanisms of gait in elderly with sporadic SVD. Previous studies have shown that global efficiency is strongly associated with cognitive function in SVD (Lawrence et al., 2014, Tuladhar et al., 2016). Here, we extend this knowledge by showing that global efficiency is also associated with gait performance in patients with sporadic SVD. In addition, this association is independent of conventional MRI markers for SVD, which are known to be related to gait impairment (de Laat et al., 2011a, de Laat et al., 2011b, de Laat et al., 2010) A possible explanation for this observation might be that these spatially distributed lesions (i.e. SVD MRI markers) predispose the brain to the disruption of white matter network (Ter Telgte et al., 2018). The disrupted network might on its turn lead to gait impairment, since gait relies on widespread connected cerebral networks (de Laat et al., 2010). In our mediation framework, the effect between network disruption and gait is mainly driven by cognitive process in elderly with SVD. The probable mechanism underlying this mediation framework could be that the structural network measures can capture both MRI-visible brain vascular injury (i.e., conventional SVD markers) and MRI-invisible injury, for example, disrupted integrity in the normal-appearing white matter. Structural network efficiency might therefore serve as an integrated and sensitive marker of SVD (Ter Telgte et al., 2018). The strong relationship between network efficiency and cognition/gait in our study suggests that cognition and gait might be affected by these underlying pathologies captured by structural network efficiency, while the involved cognitive function can further exert a cognitive effect on gait.

We found global cognitive function such as psychomotor speed and executive function to be related to gait performance, indicating gait is regulated by cognition. Of note, these two specific domains, which are among the primarily affected cognitive domains in elderly with SVD (Lawrence et al., 2014, Tuladhar et al., 2017), had a stronger association with gait disturbance relative to other domains. One prior study also found that psychomotor speed and executive control processes were important, although not exclusive, predictors of gait performance in aged population (Holtzer et al., 2006). Besides, gait variability has been taken as a sensitive marker of dynamic gait stability. We found an effect of cognitive function on gait variability domain and this was in line with previous findings showing that gait variability was associated with poor cognition (Jayakody et al., 2020, Lo et al., 2017). While gait variability was not associated with structural network efficiency.

We have identified a widely distributed subnetwork associated with stride length and cognition respectively, both with dominant connections involving the frontal lobe. The subnetwork associated with stride length disappeared after adjusting for cognitive index, corroborating the previous finding that the relation between identified subnetwork and gait is driven by cognition (Sahyoun et al., 2004) These frontal dominant edges for gait are responsible for top-down regulation of higher cognitive function, such as cognitive control, initiation, planning and regulation of motor function (Miller and Cohen, 2001). The dominant edges concerning frontal tracts provide evidence that frontal executive function plays a crucial role in gait (Sahyoun et al., 2004). For instance, deficits in the information processing from the temporal and parietal cortex to the frontal cortex might give rise to errors in anticipatory postural adjustment, resulting in gait difficulty (Takakusaki, 2017). This is especially true in older people with impaired cognitive functions. In summary, these findings are in line with emerging evidence suggesting that gait is to a certain extent a cognitive process (Amboni et al., 2013, Montero-Odasso et al., 2012, Öhlin et al., 2020).

The findings of the present study are in contrast with a recent study in young patients with CADASIL (a genetically defined SVD), in which the authors found no association between cognitive performance (i.e. processing speed) and gait (Finsterwalder et al., 2019). Among the crucial differences in that study compared to ours are that CADASIL patients only had minor gait impairment (despite a high burden of SVD), were younger and had considerably less age-related comorbidities, such as sarcopenia and joint problems. Results from this CADASIL study and other findings suggest that the effect of cognition on gait becomes more prominent in the presence of these (subclinical) comorbidities (Bridenbaugh and Kressig, 2015, Finsterwalder et al., 2019). Thus, one can speculate that one function of cognition in gait performance is to compensate for the effect of age-related comorbidities on gait in elderly.

Major strengths of the present study include the large single-center design, the inclusion of multiple MRI markers of SVD, the quantitative measurement of gait. Furthermore, all imaging data were analyzed by raters blinded to clinical information.

However, several methodological issues and limitations should be considered. First, structural networks were reconstructed from deterministic diffusion tensor tractography. The simple tensor model and fiber assignment by the continuous tracking (FACT) streamlining algorithm has limitations, including the limited ability to detect long fibers and the inability to resolve crossing fibers (Zalesky and Fornito, 2009). Nevertheless, this streamlining algorithm is a computationally inexpensive and robust method for identifying major white matter tracts (Mori and van Zijl, 2002). Second, the parcellation of brain regions might affect obtained network properties (i.e. nodes, edges). As in most previous studies, we used the AAL atlas that comprises differently sized anatomical regions to parcel brain regions for network construction. Defining the brain regions remains a challenge and alternative techniques, such as high-resolution parcellation scheme, may improve the study interpretation. Third, the cross-sectional nature of this study prevents us from making causal inference. Further research is needed to investigate the association between changes in structural network efficiency and gait decline. Fourth, we tried to exclude as many as possible patients with gait impairments other than SVD (i.e., parkinsonism, polyneuropathy, etc.). However, other mild gait impairments, like sarcopenia or degenerative musculoskeletal impairments could have been present in the older population and may have affected gait performance. Besides, the cerebellum is vital for posture-gait control, such as coordinating postural responses during walking (Takakusaki, 2017). We did not take into account the role of cerebellum in this study since DTI-based white matter network analysis is primarily applied for connectivity in supratentorial brain regions. Last but not least, cortical thinning (i.e., atrophy) was found to be related to cognitive impairment (Righart et al., 2013) and worse spatial–temporal gait performance(de Laat et al., 2012, Jayakody et al., 2020, Kim et al., 2016), while this has not been examined in the present study. Therefore, further investigation on whether if cortical atrophy could exert the effect on gait via cognitive decline is of great interest.

In conclusion, by applying network analysis based on diffusion tensor MRI, we showed that global network efficiency is associated with gait performance in SVD, which is mediated by cognitive function. Our study supports the view that cognitive function can be a mechanistic link between structural network efficiency and gait performance. This indicates that network disruption might play a crucial role for gait disturbances via cognitive dysfunction in elderly with sporadic SVD. Gait is a cognitive process to some extent and the crucial role of cognition should be taken into account when investigating gait disturbances in the elderly with SVD.

## Funding statement

M.C is supported by China Scholarship Council (201706100189). A.M.T is supported by the Dutch Heart Foundation (grant 2016 T044) and supported by the Netherlands CardioVascular Research Initiative: the Dutch Heart Foundation (CVON 2018–28 & 2012–06 Heart Brain Connection), Dutch Federation of University Medical Centers, the Netherlands Organisation for Health Research and Development and the Royal Netherlands Academy of Sciences. F-E.d.L is supported by a clinical established investigator grant of the Dutch Heart Foundation (grant 2014T060) and by a VIDI innovational grant from The Netherlands Organization for Health Research and Development (ZonMw grant 016.126.351). M.D is supported by the Radboud Excellence Initiative (18U.018651).

## CRediT authorship contribution statement

**Mengfei Cai:** Conceptualization, Methodology, Software, Formal analysis, Writing - original draft. **Mina A. Jacob:** Data curation, Writing - original draft, Writing - review & editing. **David G. Norris:** Writing - review & editing. **Marco Duering:** Writing - review & editing. **Frank-Erik Leeuw:** Conceptualization, Writing - review & editing, Funding acquisition. **Anil M. Tuladhar:** Conceptualization, Methodology, Writing - review & editing, Supervision.

## Declaration of Competing Interest

The authors declare that they have no known competing financial interests or personal relationships that could have appeared to influence the work reported in this paper.
