# Supplementary material for: Cognition mediates the relation between structural network efficiency and gait in small vessel disease
Source: Neuroimage Clin. 2021 Apr 20;30:102667. doi: 10.1016/j.nicl.2021.102667 (PMC8082689; doi:10.1016/j.nicl.2021.102667)
Supplement: Supplementary data 1 [file mmc1.docx]

**Supplementary Data** for Cai et al. Cognition mediates the relation between structural network efficiency and gait in small vessel disease.

**Supplementary Table 1. Description calculating compound scores for cognitive domains**.

| Cognitive index | Calculated as the mean of the z-scores of the Speed-Accuracy Trade-Off (SAT) score of the 1-letter subtask of the Paper-Pencil Memory Scanning Task, the mean of the Symbol-Digit Substitution Task, the mean of the SAT score of the reading task of the Stroop test, and the mean of the added score on the three learning trials and the mean of the delayed recall of the Rey Auditory Verbal Learning Test |
| --- | --- |
| Psychomotor speed | Calculated as the mean of the z-scores of the SAT score of the 1-letter subtask of the Paper-Pencil Memory Scanning Task, the mean of the SAT score of the reading subtask of the Stroop test and the mean of the Symbol-Digit Substitution Task. |
| Executive function | Calculated using the verbal fluency task, the Verbal Series Attention Test, and the interference score of the Stroop Test, which was calculated by dividing the color-word task by the mean of the reading and color naming tasks of the Stroop Test |
| Attention | Calculated as the compound z-score of the SAT score of the total time of the Verbal Series Attention Test |
| Fluency | Calculated as the mean of the z-scores of both verbal fluency tasks |
| Visuospatial memory | Calculated from the mean z-score of the mean of the immediate recall and the delayed recall trial of the Rey’s Complex Figure Test |
| Verbal memory | Calculated from the mean z-scores of the total correct words on the three learning trials and the delayed recall of the Rey Auditory Verbal Learning Test |

**Supplementary Figure 1. Flowchart of the study sample**


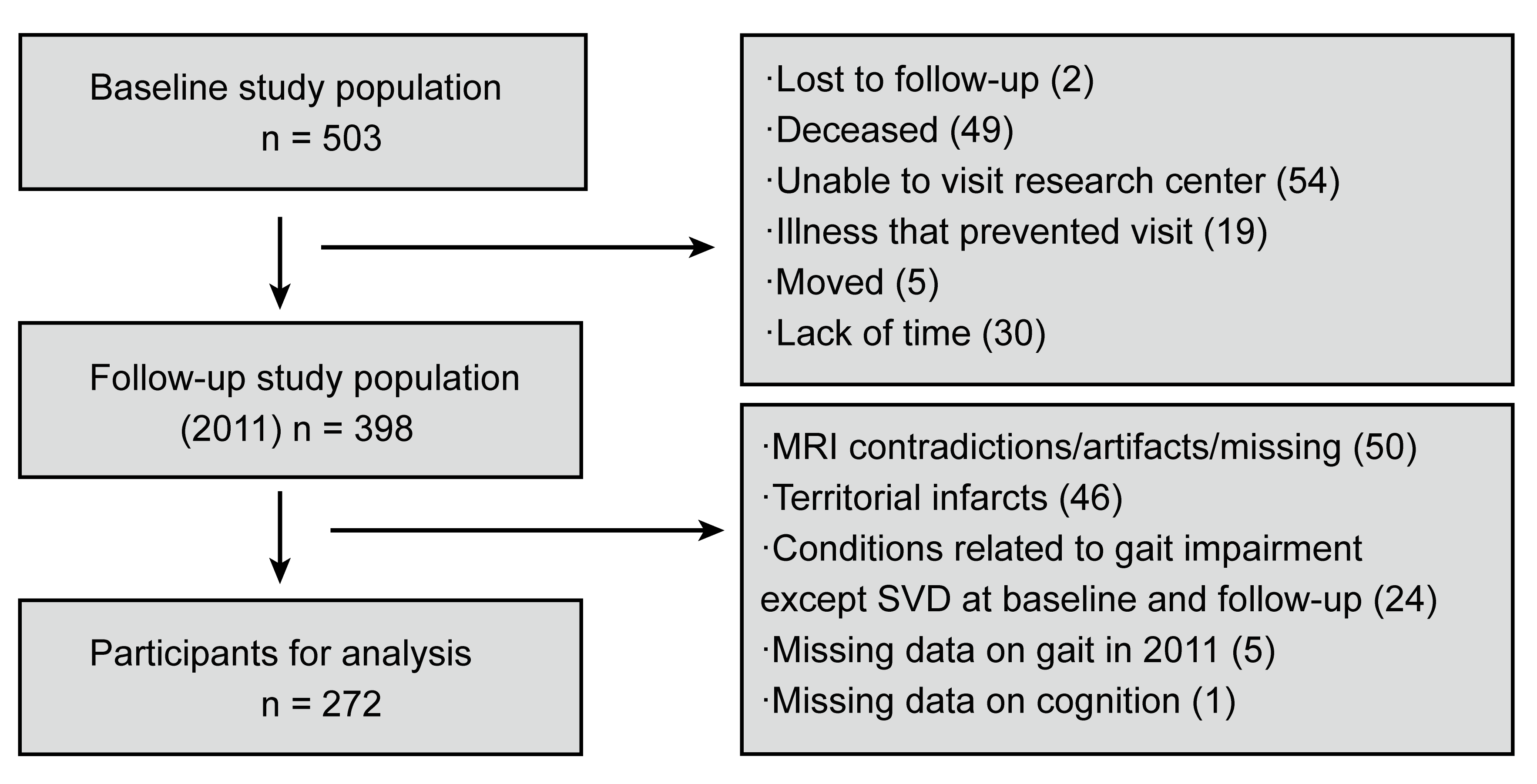


Of the 503 baseline patients in 2006, 2 patients were lost to follow-up, 49 patients had died and 54 were not able to visit our research center due to various reasons stated in the figure above, but their clinical end points were all retrieved. A total of 398 patients participated in the follow-up assessment in 2011. In this present study we additionally excluded 126 patients because of 1) missing MRI data and/or MRI artefacts (n=50), 2) territorial infarcts present on baseline and/or follow-up imaging (n=46), as these infarcts are considered potential confounders on gait performance, 3) gait related disorders other than SVD (n=24) (e.g. polyneuropathy, arthrosis in lower extremities, musculoskeletal constraints, lumbar disc herniation, parkinsonism), 4) missing gait data on follow-up (n=5) and 5) missing data on cognition during follow-up (n=1). A final sample of 272 patients was yield for analyses.
